# Insight on Tafel slopes from a microkinetic analysis of aqueous electrocatalysis for energy conversion

**DOI:** 10.1038/srep13801

**Published:** 2015-09-08

**Authors:** Tatsuya Shinagawa, Angel T. Garcia-Esparza, Kazuhiro Takanabe

**Affiliations:** 1 Division of Physical Sciences and Engineering, KAUST Catalysis Center (KCC), King Abdullah University of Science and Technology (KAUST), 4700 KAUST, Thuwal, 23955-6900, Saudi Arabia

## Abstract

Microkinetic analyses of aqueous electrochemistry involving gaseous H_2_ or O_2_, i.e., hydrogen evolution reaction (HER), hydrogen oxidation reaction (HOR), oxygen reduction reaction (ORR) and oxygen evolution reaction (OER), are revisited. The Tafel slopes used to evaluate the rate determining steps generally assume extreme coverage of the adsorbed species (*θ* ≈ 0 or ≈1), although, in practice, the slopes are coverage-dependent. We conducted detailed kinetic analyses describing the coverage-dependent Tafel slopes for the aforementioned reactions. Our careful analyses provide a general benchmark for experimentally observed Tafel slopes that can be assigned to specific rate determining steps. The Tafel analysis is a powerful tool for discussing the rate determining steps involved in electrocatalysis, but our study also demonstrated that overly simplified assumptions led to an inaccurate description of the surface electrocatalysis. Additionally, in many studies, Tafel analyses have been performed in conjunction with the Butler-Volmer equation, where its applicability regarding only electron transfer kinetics is often overlooked. Based on the derived kinetic description of the HER/HOR as an example, the limitation of Butler-Volmer expression in electrocatalysis is also discussed in this report.

Electrocatalysis has attracted tremendous attention worldwide as a sustainable and efficient energy system. Promising applications of electrocatalysis for energy conversion are electrolysis and fuel cells. Water can be electrocatalytically broken down to hydrogen (hydrogen evolution reaction, HER)^1^,^2^,^3^,^4^,^5^,^6^,^7^,^8^,^9^,^10^ and oxygen (oxygen evolution reaction, OER)^10^,^11^,^12^,^13^,^14^,^15^,^16^,^17^,^18^,^19^, which make up the well-known electrolysis process. Combining hydrogen and oxygen provides high efficiency electricity production by which chemical energy can be directly converted into electricity. Fuel cells use this process, in which hydrogen is oxidized (hydrogen oxidation reaction, HOR)^20^,^21^,^22^,^23^,^24^,^25^ and oxygen is reduced (oxygen reduction reaction, ORR)^26^,^27^,^28^,^29^,^30^,^31^,^32^,^33^,^34^,^35^,^36^,^37^. Both technologies play a crucial role in the future of sustainable societies, and thus huge research efforts have been dedicated to improving the electrocatalytic activity of these reactions—HER, HOR, ORR and OER.

In the field of electrochemistry^38^, the electric currents are experimentally measured by applying a potential to electrodes. The electric currents are proportional to the reaction rate over the electrodes. The electrocatalytic reaction rate is potential-dependent, indicating that the rate constant is also potential-dependent. Electrocatalytic reactions are generally composed of a number of elementary steps, and the forward and backward reaction rates of each electrochemical elementary step are potential-dependent. The potential-dependent nature of the electrocatalytic reaction rate is associated with the potential-dependent coverage of the intermediate species (*θ*), which is related to their formation and consumption rates.

To compare the electrocatalytic activity and to elucidate the reaction mechanism of electrocatalysts, a Tafel analysis is generally utilized. In this method, the sensitivity of the electric current response to the applied potential (Tafel slope) is analyzed, which provides information associated with the rate determining steps. The experimentally observed Tafel slopes can be compared with the theoretically derived slopes assuming different rate-determining steps based on the microkinetic model. Because its derivation process is generally complicated, the surface coverage of the intermediate species is typically assumed as constant: either *θ* ≈ 0 or *θ* ≈ 1. This simplification makes it easier for the electrochemist to consider the surface kinetics, and, in many studies, Tafel slopes derived by this method are used^39^,^40^,^41^. As previously stated, the coverage should actually vary with the applied potential: the simplification leads to an incomplete description of the actual surface kinetics that depends on the coverage. Furthermore, this assumption of invariable coverage may be applicable for steady-state conditions at constant potential and current conditions; nevertheless, for the Tafel analysis the applicability of such assumption intrinsically involves questionable accuracy. In some studies, a potential-dependent change in the Tafel slope is considered for each reaction (see refs 8, 42, 43 for the HER 21, for the HOR 44, 45, for the ORR and 46 for the OER), although in these cases, the potential and coverage were either described in insufficient detail or the Tafel slopes were conjugated according to the Butler-Volmer equation, which does not fully account for the coverage terms^5^,^9^,^31^,^47^,^48^,^49^,^50^,^51^.

This report addresses the theoretical description of the kinetics of these fundamental reactions (HER, HOR, ORR and OER) simply based on microkinetic analyses. Our aims were (1) to describe the dependence of the Tafel slope on the coverage of the formed surface species, e.g., M–H for the HER/HOR, M–OH, M–O, M–OOH and M–OO^−^ for the ORR/OER, where M is the surface site, and (2) to address the applicability of the Butler-Volmer equation in describing electrocatalytic kinetics. The visualization of the electrocatalytic kinetics, i.e., the Tafel slope dependence on coverage, provides the fundamental understanding of the potential-dependent shift in the Tafel slopes associated with the reaction mechanism changes relevant to water electrolysis and fuel cells.

## Results and Discussion

Conventionally, the Tafel analysis leads to two important physical parameters: the Tafel slope and the exchange current density. Empirically, the following Tafel relation has been well confirmed:


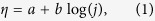


where *η* defines the overpotential, which is the difference between the electrode and standard potentials (*η* = *E* *−* *E*_*0*_), *j* denotes the current density, and *b* is the Tafel slope. Theoretically, *simple* electrochemical redox reactions can be described by the Butler-Volmer equation^52^:


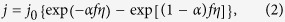


where *α* is the transfer coefficient, *f* denotes *F/RT* (*F*: the Faraday’s constant, *R*: the universal gas constant, *T*: the absolute temperature), and *j*_0_ is the exchange current density. The equation represents the total currents from both reduction and oxidation reactions (opposite signs). First, we consider only forward (or backward) rates that are sufficiently larger than the corresponding backward (or forward) reaction rate. From the above equation, the following equation can be derived:


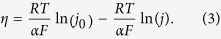


The first term in Equation 3 corresponds to *a* in Equation 1, indicating that the intercept obtained from the plot of *η* vs. log *j* can be converted into the exchange current density. The Tafel slope provides insight into the reaction mechanism, and the exchange current density is known as a descriptor of the catalytic activity^1^,^53^,^54^. Thus, for analyzing electrochemical performances, the Tafel analysis is conjugated with the Butler-Volmer equation in many studies. As described in the Introduction, the Tafel slope can be used to address the elementary steps and the rate determining steps. In the following four sections, the Tafel slope is discussed based purely on theoretical microkinetic analyses for the hydrogen evolution reaction (HER), the hydrogen oxidation reaction (HOR), the oxygen reduction reaction (ORR) and the oxygen evolution reaction (OER). Then, the fifth section discusses the rate determining steps of the opposite directions in an oxidation-reduction couple, e.g., HOR and HER. Due to the different rate-determining steps for the respective reactions, a good HOR or OER catalyst may not be a good HER or ORR catalyst, respectively. Therefore, the Butler-Volmer equation is limited to describing chemically reversible electrocatalytic reactions.

The constants include Faraday’s constant, *F* = 96500 C mol^−1^, the gas constant, *R* = 8.314 J mol^−1^ K^−1^, temperature *T*, the electron transfer coefficient, *α* = 0.5, and the surface area of the electrode, *A*.

### Hydrogen evolution reaction (HER)

The HER is generally described in two ways. The first is hydronium ion reduction,


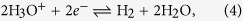


and the other is water reduction,


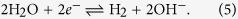


A theoretical kinetic description for each reaction is discussed in the following sections.

#### Hydronium ion reduction

Hydronium ion reduction consists of three steps: the Volmer, Heyrovsky and Tafel steps, as follows^5^,^6^,^7^,^8^,^42^,^55^,^56^.


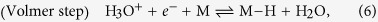



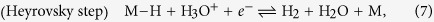



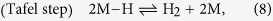


where M denotes the surface empty site. Each step can determine the overall rate, and therefore, we developed three different kinetic expressions in this section.

When the Volmer step determines the rate, the other steps should not be considered. The forward reaction rate in Equation 6,


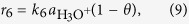


determines the HER rates. Here, *r*_*i*_ and *k*_*i*_ are the reaction rate and rate constant for *i*th Equation, and 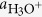 and *θ* denote the hydronium ion activities and the surface coverage by the hydrogen atom, respectively. Because this step is an electron transfer step, the kinetic rate constant depends on the applied potential, as follows:


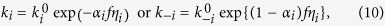


where *k*^*0*^ defines the standard rate constant for *k*, *α* is the electron transfer coefficient, *f* denotes *F/RT*, and *η*_*i*_ defines the electrode and standard potential differences (overpotential, *E* − *E*_0_) for *i*th Equation. The assumption that the Volmer step completely determines the overall rate leads to faster consumption of adsorbed hydrogen, indicating that the surface coverage should be close to 0. Therefore, using Equations 9 and 10, the reaction rate can be described by the following equation:


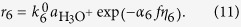


An electric current is correlated with the reaction rate according to the following equation:


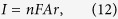


where *I* is the electric current, *n* is the number of electrons involved, and *A* denotes the surface area of the electrocatalyst. Equations 11 and 12 lead to the following expression for the electric current:


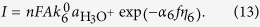


When the Heyrovsky step is the rate determining step, the adsorbed hydrogen, the reactant for the Heyrovsky step, should be taken into consideration in this case. The forward and reverse reactions of Equation 6 are pre-equilibrated in this case. The reverse reaction rate given by


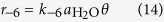


is the same as the forward reaction rate (Equation 9), resulting in the following coverage description:


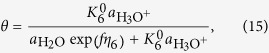


where *K*_*i*_ defines the ratio *k*_*i*_/*k*_*−i*_. The forward reaction rate for Equation 7 is calculated by the following equation:


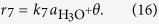


Combining Equations 10, 15 and 16, we obtain the following electric current description:


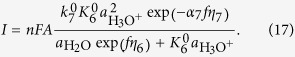


In the case of the Tafel step determining the overall rate, the reactant for this step is provided by Equation 6, indicating that Equation 15 is also valid in this case. The forward reaction in Equation 8 determines the overall rate,


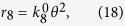


resulting in the following electric current description.


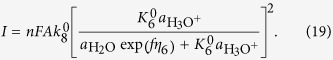


Notably, the Tafel step is not an electron transfer, so the potential dependence of the currents originates from that of the coverage.

#### Water reduction

Like hydronium ion reduction, water reduction is described by the following three steps:


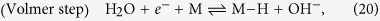



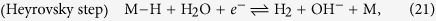


and Equation 8 for the Tafel step. In the following section, three cases involving these steps determining the rate are considered.

When the Volmer step is rate determining step, the forward reaction rate for Equation 20,


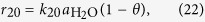


corresponds to the overall rate. As discussed in the section for hydronium ion reduction, the surface coverage can be close to zero. Therefore, combining Equations 10 and 22 yields the following current expression:


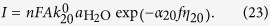


In the case of the Heyrovsky step determining the rate, the Volmer step can be pre-equilibrated, so the reverse reaction rate for Equation 20,


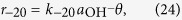


is the same as Equation 22, resulting in the following coverage description:


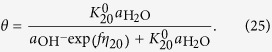


The forward reaction rate in the Heyrovsky step is given as follows:


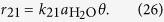


Equations 10, 25 and 26 lead to the following description of the electric currents.


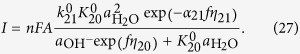


When the Tafel step determines the rate, as in the previous case, the Volmer step is pre-equilibrated, and therefore Equation 25 is valid. Because the forward reaction rate in Equation 8 is given by Equation 18, the electric current is described as follows:


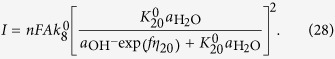


### Simulated Tafel relation for the HER

**Figure 1:**
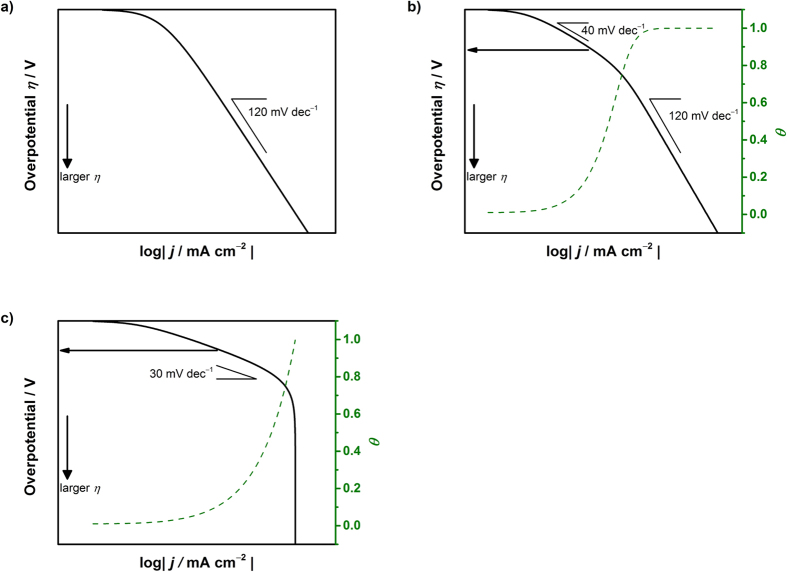
Simulated behavior of the Tafel relation for the hydrogen evolution reaction assuming (a) Equation 13 (or 23), (b) Equation 17 (or 27) and (c) Equation 19 (or 28) as the rate-determining step.

Based on these expressions, a simulation of the Tafel relations can be attempted by arbitrarily and numerically inputting the rate constants. Tafel plots for the case of *p*_H2_ = 1, *k*_3_/*k*_4_/*k*_5_/*k*_17_/*k*_18_ = 10^4^/1/10^7^/1/1 and 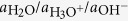 = 1/1/1 are shown in Fig. 1a,b,c for Equation 13 (or 23), Equation 17 (or 27) and Equation 19 (or 28), respectively. The objective of this study was to elucidate the Tafel slope dependence on the coverage, where hydronium ion reduction and water reduction cannot be differentiated from one another. As widely accepted, Tafel slopes of 120, 40 and 30 mV dec^−1^ were observed for the Volmer, Heyrovsky and Tafel determining rate steps, respectively, confirming the validity of our kinetic model. Additionally, for the Heyrovsky rate determining step, a Tafel slope of 120 mV dec^−1^ was observed in the higher coverage region (*θ*_H_ > 0.6). Therefore, a Tafel slope of 120 mV dec^−1^ cannot be due only to the Volmer step; it must originate from either the Volmer rate determining step or the Heyrovsky rate determining step with high adsorbed hydrogen atom coverage.

In the literature, various Tafel slopes have been reported. Pt electrocatalysts supported on carbon (Pt/C), one of the most studied catalysts, exhibits a Tafel slope of 30 mV dec^−1^ in 0.5 M H_2_SO_4_^41^,^57^,^58^, 120 mV dec^−1^ under polymer electrolyte membrane fuel cell (PEMFC) conditions^9^ and 125 mV dec^−1^ in 0.5 M NaOH solution^8^. Although this difference could be assigned to the difference in the pH levels of the solutions, in the reported study, the potential region used to obtain the Tafel slope was different: 30 mV dec^−1^ is taken at the lower overpotential range, whereas a wider overpotential range is considered for 120 mV dec^−1^. This indicates that the Tafel slope is indeed potential dependent and, in turn, coverage dependent. In other reports that focused on Pt electrocatalysts, the Tafel slope of bulk Pt disk electrodes is reported to exhibit potential dependence: 36–68 mV dec^−1^ followed by 125 mV dec^−1^ with an increasing overpotential in a 0.5 M H_2_SO_4_ electrolyte solution^8^. The electrocatalytic activity of Pt toward the HER is known as structure sensitive, as different facets shows various activities and rate determining steps^59^,^60^. The Tafel slope for Pt(110) is two-step, starting from 55 mV dec^−1^ shifting to 150 mV dec^−1^, Pt(110) exhibits a slope of 75 mV dec^−1^ that shifts to 140 mV dec^−1^, and Pt(111) is reported to exhibit a Tafel slope of 140–150 mV with no transition in a 0.1 M KOH solution^59^. These examples demonstrate the significance in considering potential-dependent Tafel slopes.

Other electrocatalysts in addition to Pt have also been reported to exhibit different Tafel slopes. Ir/C, Pd/C and Rh/C show slopes of 124 ± 5, 127 ± 8 and 95 ± 3 mV dec^−1^, respectively, considering a wide range of overpotentials under PEMFC conditions^9^. Bare Ni, Mo, MoNi, MoNi_2_, MoNi_3_ and MoNi_4_ alloy exhibit Tafel slopes of 121–142^42^,^43^, 126, 132, 142, 148 and 138 mV dec^−1^ in 1.0 M NaOH solution, respectively^43^. Ni-Mo-Cd multi-metal electrodes present a two-step Tafel slope of 30–38 mV dec^−1^ that shifts to 125 mV dec^−1^^42^, and Pt-Ce electrodes exhibit a Tafel slope of 114 mV dec^−1^ in 1.0 M NaOH^61^. Carbon is generally used as a catalyst support, which also becomes active toward the HER by introducing foreign atoms: graphite, P-graphene, N-graphene and N,P-graphene are reported to exhibit Tafel slopes of 206, 133, 116 and 91 mV dec^−1^ in 0.5 M H_2_SO_4_ and 208, 159, 143 and 145 mV dec^−1^, respectively, in 0.1 M KOH^62^. When Pt-Pd supported on reduced graphene oxide is used, the Tafel slope is 10–25 mV dec^−1^ at an overpotential <40 mV^63^, which is lower than the afore-derived Tafel slopes. However, as shown in Fig. 1, even the theoretical Tafel slopes at smaller overpotentials do not reach the well-known values of 30, 40 and 120 mV dec^−1^ in every case. Therefore, if too small an overpotential region is considered, the Tafel analysis leads to a misrepresentation of the rate-determining step and furthermore poorly compares to other reported values.

In addition to pure metal and bimetallic electrocatalysts, sulfide^41^,^64^,^65^, phosphide^57^,^58^,^66^,^67^ and nitride^68^ have also been studied. MoS_2_ exhibits 94–110 mV dec^−1^ in 0.5 M H_2_SO_4_ electrolyte solution^41^,^64^ and 55–60 mV dec^−1^ in H_2_SO_4_ at pH 0.24^65^. These values decrease to 41 mV dec^−1^ when reduced on graphene oxide^41^ or to 43 mV dec^−1^ due to treatment with n-BuLi^64^. In 0.5 M H_2_SO_4_ solution, CoP^57^ and NiP^58^ exhibit slopes of 50 and 75 mV dec^−1^, respectively. Under other conditions, Ni_2_P in 1.0 M H_2_SO_4_ exhibits a Tafel slope of 60 that steps to 136 mV dec^−1 ^67, which suggests that for phosphide, the Tafel slope is also potential-dependent. The Tafel plots for MoP and MoP|S (MoP with a phosphosulfide surface) exhibit slopes of 50 mV dec^−1^ that increases with potential in 0.5 M H_2_SO_4_^66^. Even for the nitride, the Tafel slope increases with the overpotential for use of δ-MoN and Co_0.6_Mo_1.4_N_2_ in 0.1 M HClO_4_^68^. These studies support the importance of evaluating in detail the theoretical Tafel slope as potential-dependent.

### Hydrogen Oxidation Reaction (HOR)

The HOR can be described in two ways,


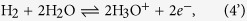



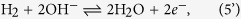


which are the reverse expressions of the HER. Hydrogen can be oxidized either by a water molecule or a hydroxide anion. In this section, the HOR is described in both cases.

#### HOR with water molecule

The HOR with a water molecule is the reverse reaction of hydronium ion reduction, and the elementary steps are described using the same equations as Equations 6, 7, 8 20, 21, 22,


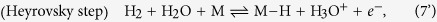



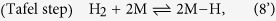



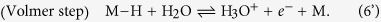


Each case determining the overall reaction rate is discussed in the following sections.

When the Heyrovsky step determines the rate, the adsorbed hydrogen is consumed via the Volmer step. In addition, it is assumed that the Tafel step does not occur or is slow and therefore negligible. The forward reaction rate in Equation 7’ is described by the following equation:


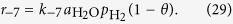


The subscript has a negative value to show similar applicability to the HER. Assuming that the Heyrovsky step determines the overall rate, the consumption of adsorbed hydrogen is faster than its formation, and the surface coverage is close to zero. Combining Equation 29 with Equations 10 and 12 yields the following expression for current:


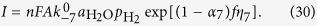


When the Tafel step is the rate determining step, similar to the previous case, it is assumed that the Heyrovsky step is slow and that the coverage is close to zero. Thus, the rate expression for the Tafel step,


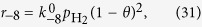


yields the following electric current:


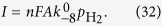


In this case of the Volmer step determining the rate, the reactant is provided by two reactions: the Heyrovsky step and the Tafel step. We separately address the two types of adsorbed hydrogen atoms as those formed via the Heyrovsky step (*θ*_1_) and those formed via the Tafel step (*θ*_2_). The coverage of *θ*_1_ is given by considering the pre-equilibrium of Equation 7’. The reverse reaction rate is described by Equation 16, which is equilibrated with Equation 29 to yield the following coverage expression:


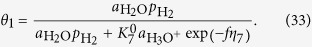


Notably, *K*_*i*_ defines the ratio of *k*_*i*_/*k*_*−i*_ (not *k*_*−i*_/*k*_*i*_) to be consistent with the HER section. The other coverage expression can be obtained by considering the equilibrium of Equations 18 and 31, as follows:


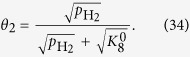


The parameter *ε*_*i*_ (0 < *ε* ≤ 1, ∑(*ε*_*i*_*θ*_*i*_) ≤ 1) is introduced to describe the difference in the practical reactivity of adsorbed hydrogen. If the ratio of *ε*_1_/*ε*_2_ is unity, there is no difference between the two types of adsorbed hydrogen atoms. The conditions *ε*_1_ « *ε*_2_ and *ε*_2_ « *ε*_1_ correspond to the Tafel-Volmer and Heyrovsky-Volmer steps, respectively. The forward reaction rate in Equation 6’ is described by Equation 11, and, by combining Equations 10, 14, 33 and 34, we obtain the following electric current expression,


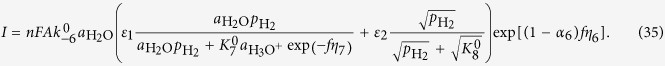


#### HOR with hydroxide anions

The elementary steps are given by the following equations:


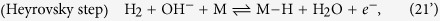



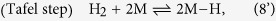



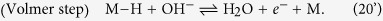


When the Heyrovsky step is rate determining, the surface coverage is close to zero, and the Volmer step is negligibly slow. The forward reaction rate for Equation 21’ is described by the following equation:


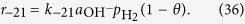


The assumptions made here and in Equations 10 and 36 give us the following current expression:


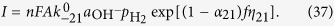


In the case of the Tafel step determining the rate, the Volmer step description for the HOR with hydroxide anion is equivalent to that for the HOR with water. Therefore, Equation 32 also gives the electric current for this case.

When the Volmer step is the rate determining step, similar to the previous cases for the HOR with water, we separately address two types of adsorbed hydrogen atoms. The hydrogen adsorbed via the Volmer step is described by Equation 33. The other coverage can be expressed by considering the pre-equilibrium phase of Equation 21’. The reverse reaction rate is given by Equation 26, which is equilibrated with the forward reaction rate of Equation 36. Thus, the following coverage expression, *θ*_3_, for adsorbed hydrogen via Equation 18 is obtained:


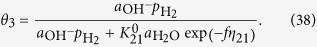


Using *ε*_*i*_, the electric current for this case is described by the following equation:


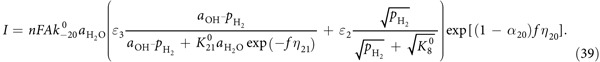


#### Simulated Tafel relation for the HOR

**Figure 2:**
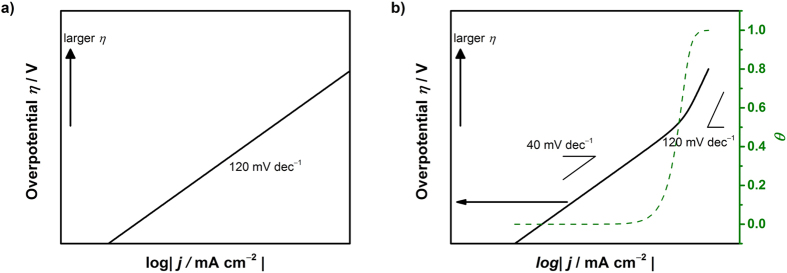
Simulated behavior of the Tafel relation for the hydrogen oxidation reaction assuming (a) Equation 30, 35 (K_7_ < 10^15^), 37 or 39 (K_21_ < 10^–15^ and pH 13) and (b) Equation 35 or 39 as the rate-determining step.

Based on these expressions, Tafel relations are simulated using *k*_3_/*k*_4_/*k*_5_/*k*_17_/*k*_18_ = 1/1/1/1/1 and 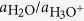 = 10/1 or 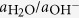 = 10/1 in Fig. 2. Notably, Equation 32 (reaction rate is completely determined by the Tafel step) yields a constant current independent of the potential. In Fig. 2a, both Equations 30 and 37 can be depicted with a simulated Tafel slope of 120 mV dec^−1^ (Heyrovsky step determining the rate). Similarly, for Equations 35 and 39, the simulated current can be described as shown in Fig. 2a for specific cases (*K*_7_ < 10^15^ for Equation 35, *K*_21_ < 10^−15^ and pH 13 for Equation 39). In other cases, for Equations 35 and 39, the simulation shows a two-step Tafel slope, as shown in Fig. 2b. The Tafel slope is 40 mV dec^−1^ with a surface coverage approaching zero (*θ*_H_ < 0.4), and 120 mV dec^−1^ is obtained with a high surface coverage (*θ*_H_ > 0.6). For the Tafel-Volmer step, when *θ*_H_ is varied from 0 to 1 by tuning *K*_*5*_, the Tafel slope is always 120 mV dec^−1^. As previously mentioned, 120 mV dec^−1^ can be observed in many cases, suggesting that the Tafel slope of 120 mV dec^−1^ cannot be solely used to identify the rate determining step.

In the literature, Tafel slopes using noble metals and their alloys are used to evaluate the HOR kinetics. Tafel slopes of 106, 88, 229, 154 and 784 mV dec^−1^ were reported for bulk Pt, sputtered Pt, Pt-Ni, Pt-Ti and Ni-Ti, respectively, in 1 M KOH^69^. Tafel slopes of 124 ± 15, 124 ± 5, 258 ± 23 and 180 ± 8 were reported for Pt/C, Ir/C, Pd/C and Rh/C, respectively, where the Tafel step is mentioned as the rate-determining step^9^. The HOR is also a structure sensitive reaction: Pt(110) has a Tafel slope of 28 mV dec^−1^ (**Tafel**-Volmer), Pt(100) has a Tafel slope of 37 that increases to 112 mV dec^−1^ (**Heyrovsky**-Volmer), and Pt(111) has a Tafel slope of 74 mV dec^−1^ (Tafel-Volmer, Heyrovsky-Volmer) in 0.05 M H_2_SO_4_^70^. These examples corroborate the significance of this study: a Tafel slope of around 120 mV dec^−1^ is frequently reported but is not evidenced in any rate-determining step.

### Oxygen reduction reaction (ORR)

The ORR mechanism is complicated and continues to be discussed^26^,^28^,^40^,^44^,^47^,^48^,^71^,^72^. Recently, Adzic and coworkers reported that OO^−^ can be the intermediate species for ORR under alkaline conditions^73^. Therefore, as elementary steps, the following associative mechanism is considered in this study^26^,^27^,^28^,^29^,^30^,^31^,^74^,^75^:


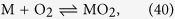



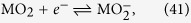



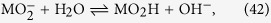



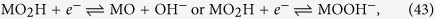


where M denotes an empty site on the surface. For acidic conditions, the following step is introduced instead of Equations 41 and 42:


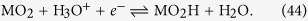


Further details of the elementary steps have been discussed by Koper, where charge transfer and proton-coupled charge transfer reactions are separately considered^76^. Although above steps are in the more simplified form, the resultant Tafel slope results in identical values. Each step of Equations 41, 42, 43, 44 is assumed to be rate determining to describe the electric currents in the following sections. Regarding the coverage expression, *θ*_0_, *θ*_1_, *θ*_2_ and *θ*_3_ denote the surface coverage by the empty site, MOO, MOO^−^ and MOOH, respectively.

#### Equation 41 determines the overall reaction rate

When the formation of MOO^−^ is the rate determining step, Equation 40 can be assumed to be at equilibrium. The forward and reverse reaction rates of Equation 40 are the same:


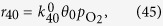



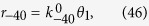


where *θ*_0_ and *θ*_1_ denote the surface coverage by the empty sites and MOO, respectively. Because Equation 41 is rate determining, the surface coverage by MOO^−^ is close to zero, yielding the following relationship:


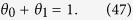


Equations 45, 46, 47 lead to the following coverage expression:


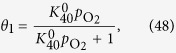


where 
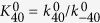
. The forward reaction rate for Equation 41 is described as


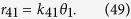


The current expression is provided as follows using Equation 10, 48 and 49,


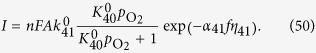


#### Equation 42 determines the overall reaction rate

In this case, Equations 40 and 41 are equilibrated. The forward and reverse reaction rates for Equation 41 are described as in Equation 49 and


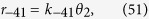


respectively, where *θ*_2_ denotes the surface coverage by MOO^−^. Because the forward reaction in Equation 42 determines the overall reaction rate, the surface adsorbed species are MOO and MOO^−^, indicating that the following relationship is true:


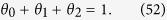


Equations 45, 46, 49, 51 and 52 lead to the following coverage expression:


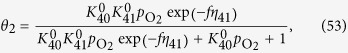


where 
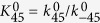
. The forward reaction rate for Equation 42 is described as


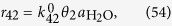


and, therefore, the overall electric current is expressed, as follows:


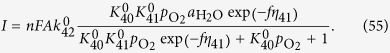


Of note, Equation 42 is not an electron transfer reaction, and therefore that the reaction rate dependence on the applied potential originates from that of the coverage.

#### Equation 43 determines the overall reaction rate

In addition to Equations 40 and 41, Equation 42 is equilibrated. The forward and reverse reaction rates for Equation 42 are described as in Equation 54 and


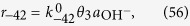


where *θ*_3_ denotes the surface coverage by MO, and the following relationship is true:


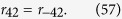


Among the three adsorbed species, the following relationship is true:


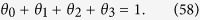


Equations 45, 46, 49, 51, 52, 54, 56, 57 and 58 lead to the following coverage expression:


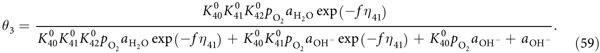


Because the forward reaction rate in Equation 43 is given as


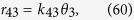


the overall reaction rate is described, as follows:


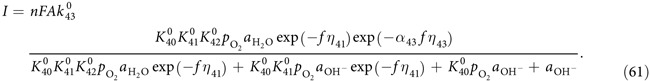


#### Equation 44 determines the overall reaction rate

Instead of Equations 40 and 41, Equation 44 is considered to be rate determining. Equations 45, 46, 47, 48 are valid in this case, and the overall rate expression is given, as follows:


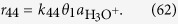


This leads to the following equation for the electric current:


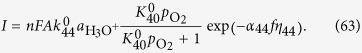


#### Simulated Tafel relation for the ORR

**Figure 3:**
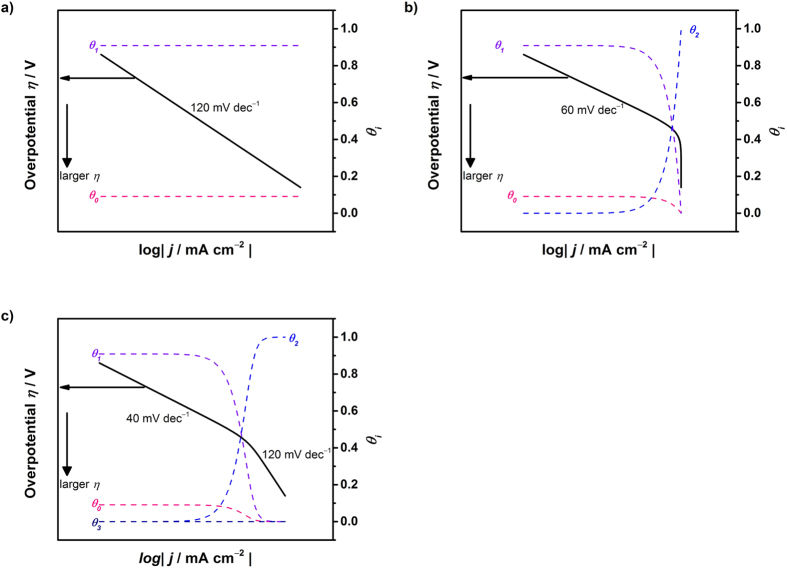
Simulated behavior of the Tafel relation for the oxygen reduction reaction assuming (a) Equation 50 or 63, (b) Equation 55 and (c) Equation 61 as the rate-determining step.

We describe the four kinetic expressions for the ORR. Using Equations 50, 55, 61 and 63, the current-potential relationships are shown in Fig. 3. The following values were used for constants: *k*_43_/*k*_44_/*k*_45_/*k*_46_/*k*_47_/*k*_−43_/*k*_−44_/*k*_−45_ = 1/1/1/1/1/1/10^5^/10^4^/10 and 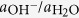 = 1/10. It should be noted that the Tafel slope itself is not affected by these figures, but the potential region where the specific Tafel slope is observed is quantitatively dependent on these values, although this falls outside the scope of our study. The primary differences between sections describing Equation 41 and 44 being rds are the constants, as can be observed in Equations 50 and 63. Therefore, both cases can be described simultaneously (Fig. 3a). Figure 3b,c describe Equations 55 and 61, respectively. The Tafel slope of 120 mV dec^−1^ is obtained when the rate is determined by the first discharge step or the upon consumption of the MOOH species with high coverage of MOO^−^ (Equation 43). In the other cases, the simulated Tafel slope is lower than 120 mV dec^−1^, as Equations 55 and 61 correspond to Tafel slopes of 60 mV dec^−1^ and 40 mV dec^−1^, respectively.

Various Tafel slopes have been reported in the literature. Pt is one of the most active electrocatalysts for the ORR and has been studied under various conditions. The Pt/C catalyst under acidic conditions exhibits a two-step Tafel slope: ca. 60 mV dec^−1^ shifts to 120 mV dec^−1^ with increasing potential in 0.5 M H_2_SO_4_, 0.05 M H_2_SO_4_ and 0.5 M HClO_4_^77^,^78^,^79^,^80^,^81^. A single Tafel slope of 50–80 mV dec^−1^ (higher with decreasing Pt mean diameter from 6 to 1 nm) has been reported in 5 mM HClO_4_, possibly measured at a lower overpotential range (corresponding to the aforementioned 60 mV dec^−1^)^82^. In an alkaline solution of 1 M NaOH, Pt/C also exhibits a two-step Tafel slope of 65–82 mV dec^−1^ that increases to >100 mV dec^−1^ with increasing overpotential^83^. Bare Pt disks exhibit similar behavior: a Tafel slope of 60 mV dec^−1^ at a low overpotential and 120 mV dec^−1^ at higher overpotentials in 0.1 M HClO_4_^31^,^44^,^84^ and in HClO_4_ or H_2_SO_4_ at pH 0.3–4^71^,^72^. When an additional supporting electrolyte is used, a single Tafel slope of 120 mV dec^−1^ is observed^85^. In addition, under PEMFC conditions, a single Tafel slope of 109–120 mV dec^−1^ at 65 °C was reported^86^. Under neutral conditions of 0.05 M K_2_SO_4_, various Tafel slopes were found: 175 mV dec^−1^ that decreased to 120 mV dec^−1^ at pH 4 and 120 → 175 → 120 mV dec^−1^ at pH 6^85^. A constant Tafel slope of 125 mV dec^−1^ was found in 10 mM NaOH + 0.1 M Na_2_SO_4_^81^. Under alkaline conditions, Pt exhibits a Tafel slope of approximately 60 mV dec^−1^ in the low overpotential region in 0.1–6.0 M KOH^49^,^50^,^69^,^87^, 1.0 M K_2_B_4_O_4_ and 1.0 M KF^28^. At larger overpotentials, the Tafel slope increases to: 120 mV dec^−1^ in 1.0 M K_2_B_4_O_4_ and 1.0 M KF^28^, 180 mV dec^−1^ in 0.01 M KOH^87^, 200–490 mV dec^−1^ in 0.1 M KOH^49^,^87^, 215–310 in 0.5 M KOH^50^,^87^, 242–270 in 1.0 M KOH^50^,^69^,^87^, and 252–300 in 3.0 M KOH^50^,^87^. Single Pt crystals exhibit different Tafel slopes depending on the exposed facet. Constant Tafel slopes of 76–77 mV dec^−1^ in 0.1 M HClO_4_^88^,^89^ and 120–130 mV dec^−1^ in 0.05–0.5 M H_2_SO_4_^90^ were found for Pt(111). However, two-step Tafel slopes were reported for Pt(100) and Pt(110). Pt(100) exhibits a Tafel slope of 100 mV dec^−1^ in 0.1 M HClO_4_^89^ or 65 mV dec^−1^ in 0.05 M H_2_SO_4_^91^ at lower overpotentials and 120 mV dec^−1^ with increasing potentials, and Pt(110) exhibits a Tafel slope of 80–82 mV dec^−1^ that increases to 110 mV dec^−1^ in 0.1 M HClO_4_ and 0.05 M H_2_SO_4_^89^,^91^. Under alkaline conditions, the Tafel slope generally increases. A constant Tafel slope of 75 mV dec^−1^ for Pt(111), a two-step Tafel slope of 86 that increases to 167 mV dec^−1^ for Pt(100), and a two-step Tafel slope of 89 that increases to 265 mV dec^−1^ for Pt(110) in 0.1 M KOH were reported^92^.

Alloying Pt with other metals is a common way to improve the ORR activities, among which Pt-Ni alloy is one of the most studied and active electrocatalysts. PtNi mesostructured thin film exhibit a Tafel slope of 40 mV dec^−1^ in 0.1 M HClO_4_^84^, Pt_7_Ni_3_ exhibits a Tafel slope of 83 mV dec^−1^ in 1.0 M H_2_SO_4_^93^, and Pt_3_Ni exhibits a Tafel slope of 46 mV dec^−1^ in 0.1 M HClO_4_^34^, whereas Pt_61_Ni_39_ was reported to show a potential-dependent Tafel slope in 0.1 M HClO_4_^94^. With other transition metals, Pt_71_Co_29_ and Pt_51_Fe_49_ exhibit comparable Tafel slopes, which increased up to 120 mV dec^−1^ in 0.1 M HClO_4_^94^, and PtCo/C presents a Tafel slope of 70 mV dec^−1^ over the entire potential range of 0.8–0.9 V vs. RHE^32^. Two-step Tafel slope values of 47 → 141, 49 → 206 and 48 → 304 mV dec^−1^ were found for Pt-Cr, Pt-Ta and Pt-Cr-Ta, respectively, in 1.0 M KOH^69^. Pt_3_Sc and Pt_3_Y also exhibit two-step Tafel slopes; at lower overpotentials, the Tafel slope is comparable to Pt and increases with the potential in 0.1 M HClO_4_^95^. PtPd alloy exhibits a comparable Tafel slope to Pt of 60 mV dec^−1^ that increases to 130 mV dec^−1^ in 0.5 M H_2_SO_4_^96^.

Regarding other electrocatalysts that do not contain platinum, Ir was reported to exhibit Tafel slopes of 60 and 120 mV dec^−1^ at low and high overpotentials, respectively, in LiClO_4_ solutions at pH values of 2.2, 3.1, and 11.0^97^. In addition to metal catalysts, some oxides (57 mV dec^−1^ for MnO_x_/C, 56 mV dec^−1^ for Ni-MnO_x_/C, 47 mV dec^−1^ for Mg-MnO_x_/C)^98^ and carbon species (110 mV dec^−1^ for pyrolytic graphite)^49^ exhibit ORR activities under alkaline conditions. In many cases, a variety of Tafel slopes are reported, and most are potential-dependent. To identify or at least consider the rate determining step, deriving the theoretical Tafel slope dependence on each coverage is of particular importance.

### Oxygen evolution reaction (OER)

The oxygen evolution reaction (OER) is known as a 2 or 4 electron step, and the reaction mechanism is complicated^39^,^56^,^99^. Based on the literature and considering that the OER with hydroxide anion is the backward reaction of the ORR, we considered the following mechanism under alkaline conditions, assuming a single-site mechanism:


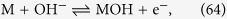



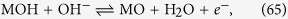



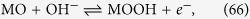



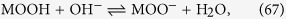



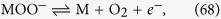


where M denotes a site on the surface. Each step of Equations 64, 65, 66, 67, 68 is assumed to be rate determining to describe the electric currents. Regarding the coverage expression, *θ*_0_, *θ*_1_, *θ*_2_, *θ*_3_ and *θ*_4_ denote the surface coverage by the empty site, MOH, MO, MOOH and MOO^−^, respectively.

#### Equation 64 determines the overall reaction rate

The reaction rate for this case is simply described as follows, assuming the coverage of the empty site (*θ*_0_) is ≈0,


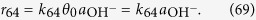


For the electron transfer oxidation reaction, the rate constant is generally described by Equation 10, yielding the following overall kinetic rate equation:


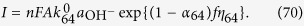


#### Equation 65 determines the overall reaction rate

In this case, the reaction given by Equation 64 can be assumed to be at equilibrium,


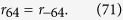


The kinetic expression of the backward reaction of Equation 64 is:


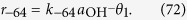


Equations 69 and 72 yield the following relationship:


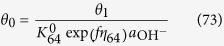


and the following limitation applies:


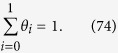


The coverage description is obtained as follows using Equations 73 and 74:


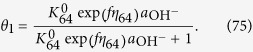


Therefore, the kinetic current is described as:


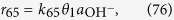



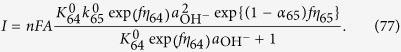


#### Equation 66 determines the overall reaction rate

The equilibrium of Equation 64 is also applies, indicating that Equation 73 is true. Additionally, Equation 65 is at equilibrium:


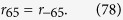


The backward reaction rate for Equation 65 can be written as:


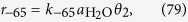


Then, the following relation for the coverage is obtained:


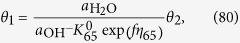


Additionally, the following is true:


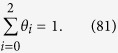


Combining Equations 73 gives the following coverage expression:


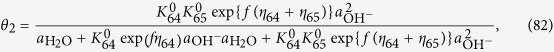


which yields the following kinetic rate equation:


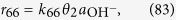



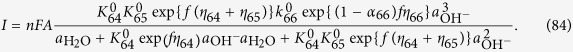


#### Equation 67 determines the overall reaction rate

This case regarded in a similar manner to the previous case. Considering equilibrium in Equation 66:


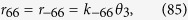


the following relation is obtained:


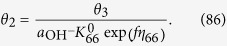


Furthermore,


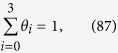


is true in this case, and therefore the following coverage expression is derived by combining Equations 73:


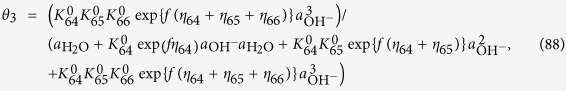


Therefore, the kinetic current is given as:


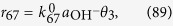



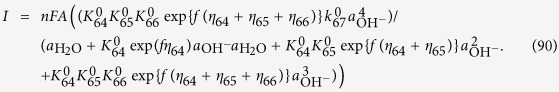


#### Equation 68 determines the overall reaction rate

Equation 67 at equilibrium corresponds to:


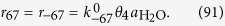


Combining with Equation 89 yields the following relation:


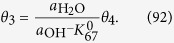


Considering that,


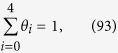


is true in this case and combining Equations 73 and 93, we obtain the following coverage expression:


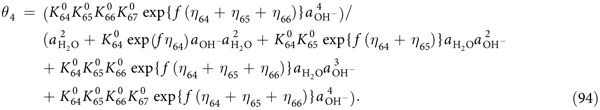


Finally, the kinetic current for this case is given as:


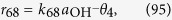



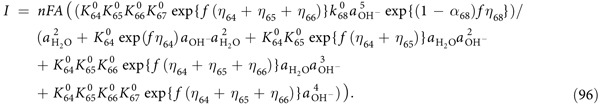


#### Simulated Tafel relation for the OER

**Figure 4:**
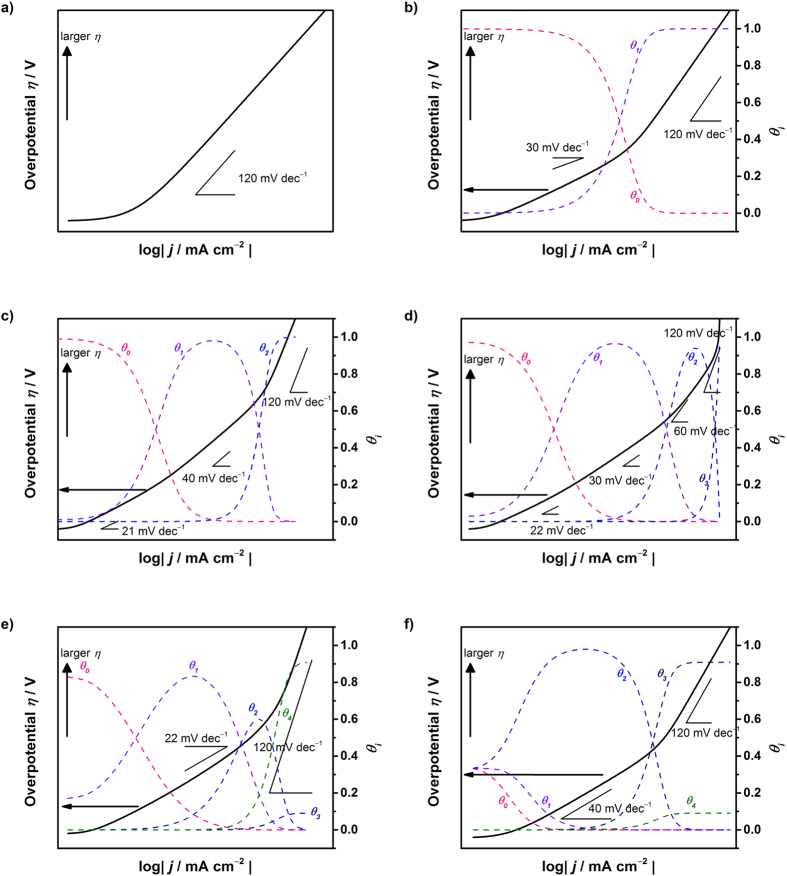
Simulated behavior of the Tafel relation for the oxygen evolution reaction assuming (a) Equation 70, (b) Equation 77 (c) Equation 84 (d) Equation 90 and (e,f) Equation 96 as the rate-determining step.

The Tafel plots for the OER can be visualized. Using Equations 70, 77, 84, 90 and 96, the current-potential relationships are shown in Fig. 4. The following values were used: 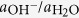 = 1/10 and

(for Equation 77) *k*_67_/*k*_68_/*k*_−67_ = 10^2^/10^7^/1: Fig. 4b

(for Equation 84) *k*_67_/*k*_68_/*k*_69_*/k*_−67_/*k*_−68_ = 10^4^/10^2^/1/10^9^/1: Fig. 4c

(for Equation 90) *k*_67_/*k*_68_/*k*_69_*/k*_70_/*k*_−67_*/k*_−68_/*k*_−69_ = 3 × 10^12^/10^6^/10^4^/1/10^11^/10^12^/10^15^: Fig. 4d

(for Equation 96)

*k*_67_/*k*_68_/*k*_69_*/k*_70_/*k*_71_/*k*_−67_*/k*_−68_/*k*_−69_/*k*_−70_ = 10^9^/10^10^/10^8^/10^11^/1/10^16^/10^16^/10^19^/10^10^: Fig. 4e

or

*k*_67_/*k*_68_/*k*_69_*/k*_70_/*k*_71_/*k*_−67_*/k*_−68_/*k*_−69_/*k*_−70_ = 10^11^/10^14^/10^8^/10^9^/1/10^14^/10^14^/10^19^/10^10^: Fig. 4f.

According to these plots, a Tafel slope of 120 mV dec^−1^ is observed when the surface species formed in the step just before the rate-determining step is predominant (e.g., *θ*_1_ for Equation 77, *θ*_2_ for Equation 84, and so on). In the other cases, the Tafel slope is lower than 120 mV dec^−1^. When the surface adsorbed species produced in the early stage of the OER remains predominant, the Tafel slope decreases. In particular, as shown in Fig. 4b, when Equation 65 determines the overall rate, a Tafel slope of 30 mV dec^−1^ was observed with high coverage of the empty site. In addition, the same Tafel slope was theoretically observed when Equation 67 was the rate determining with a high coverage of MOH (>0.6). This may account for the unusually small Tafel slope of 30 mV dec^−1^ that was observed using NiFe LDH (31 mV dec^−1^ in 1 M KOH and 35 mV dec^−1^ in 0.1 M KOH using NiFe-LDH/CNT)^13^ and when using Fe_50_Co_50_O_X_, Fe_50_Ni_50_O_X_ and Fe_33_Co_33_Ni_33_O_X_ (approximately 30 mV dec^−1^ in 0.1 M KOH)^100^ under alkaline conditions. When the NiFe LDH is used in the bulk form, the Tafel slope differs from around 30 mV dec^−1^; a Tafel slope of approximately 60–65 mV dec^−1^ was found, which was also reported using NiCo LDH and CoCo LDH^101^. These values are comparable to 65 mV dec^−1^ for Ni(OH)_2_ and 60 for Ir/C in 0.1 M KOH^12^. Other metal derived electrocatalysts of oxidized Ni, Co and Fe were reported to exhibit a Tafel slope of approximately 40 mV dec^−1^, which shifts to higher Tafel slope values with increasing overpotential (up to ca. 240 mV dec^−1^) in 0.1–5.0 M NaOH^101^,^102^. In addition, the perovskite-type catalyst, LnBaCo_2_O_5+nδ_ (Ln: Pr, Sm, Gd, and Ho), exhibited a Tafel slope of 60 mV dec^−1^ in 0.1 M KOH^103^. Tafel slopes of 292, 312 and 393 mV dec^−1^ have been reported in the literature using NiCo_2_O_4_ nanoneedles, CoPi and NiCo_2_O_4_ nanosheets, respectively, in 1.0 M KOH^104^. In acidic electrolyte solution, Tafel slopes of 41, 74, 66, 85, 210, 120 and 90 mV dec^−1^ were reported for Ru, Ru-Ir, RuO_2_/TiO_2_, Ir, Ir-Pt, Ru-Pt and Pt, respectively, in 1.0 M H_2_SO_4_ at 80 °C^105^. Regarding Pt, studies performed at room temperature reported a Tafel slope of 110 mV dec^−1^ in 1 M HClO_4_, whereas a slope of 60 that increased to 120 mV dec^−1^ with increasing potential in 1.0 M KOH^106^. In some cases, although not clearly documented, the Tafel slope changes with potential^13^,^100^,^104^,^106^. To electrochemically elucidate the rate determining step and elementary step, not only the smallest Tafel slope but also all measured Tafel slopes should be reported, whereby this study can then be used to identify the possible rate determining steps.

### Overall description of electrocatalytic reaction

#### Applicability of the Butler-Volmer equation

In the first two sections, the kinetic electric currents were derived for the HER and HOR, respectively, resulting in six types of electric current expressions for each HER and HOR. These analyses demonstrated that the HER and HOR are not a simple reversible electrocatalytic reaction pair because different rate determining steps can be involved in each. Thus, for the HOR/HER *to follow the Butler-Volmer equation, at least the same type of rate-determining steps* should be prevalent. For example, when the Volmer step (Equation 6) determines both reactions, the HER can be described by Equation 13, and the HOR can be described by Equation 35. By introducing the exchange current, *I*_*0*_, Equation 13 can be simplified as follows:


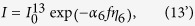


where


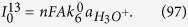


Equation 35 cannot be simplified in this way because the coverage term is potential-dependent. Only if we assume a constant coverage can we obtain


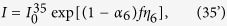


where


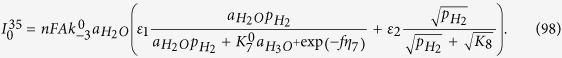


In this case, the combination of Equations 13’ and 35’ leads to the following overall HOR/HER current equation:


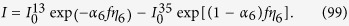


Equation 99 resembles the Butler-Volmer equation^52^:


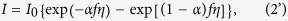


which implies the following:

{(1) if the Volmer step determines both HER and HOR}

And

{(2A) if *ε*_1_ is negligibly small compared to *ε*_2_ (Tafel-Volmer step)

Or

(2B) if *K*_*7*_ is negligibly small compared to 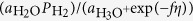 (Heyrovsky-Volmer step, and *θ*_H_ is close to 0)}

And

{(3) if the surface coverage during hydronium ion reduction remains close to zero at any potential}

And

{(4) if the surface coverage during hydrogen oxidation by water molecules remains close to unity at any potential}

then the HOR/HER may be described by the Butler-Volmer equation. Regarding point (1), assuming that the Volmer step determines the rate is difficult in practice. A Tafel slope of 120 mV dec^−1^ for the HER cannot be used as evidence for the Volmer step being the rate-determining step, as mentioned in the HER section. Additionally, as described in the HOR section, Tafel slopes that differ from 120 mV dec^−1^ for the HOR can be theoretically obtained only if adsorbed hydrogen species are formed via the Heyrovsky step and if the surface coverage *θ*_H_ is close to 0. These rationales suggest that there are other scenarios in which the Tafel slope can be 120 mV dec^−1^. Hence, an experimentally observed slope of 120 mV dec^−1^ does not imply that the Volmer step limits the rate. Furthermore, to meet criteria (3) and (4), there should be a certain potential range where coverage changes with potential, which contradicts the criteria themselves. Thus, the assumption that the Volmer step determines the overall rate at any potential is not true in any case. If all of the above criteria are satisfied, then the Butler-Volmer equation may be applied to fit the HOR/HER. Otherwise, the obtained fitting parameter may result in misleading information.

Nevertheless, in some studies, the Butler-Volmer equation^52^:


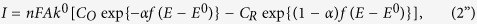


is applied to elucidate the surface kinetics^5^,^9^,^31^,^47^,^48^,^49^,^50^. The empirical Tafel equation (Equation 1) can be simplified to the equation based on the Butler-Volmer equation (Equation 3). This conversion of the equation is valid *only if* the Butler-Volmer equation is applicable. Even for the HOR/HER, one of the simplest electrochemical reactions, the Butler-Volmer equation is applicable only in very limited cases. Notably, the Butler-Volmer equation is derived assuming “*the special case in which the interface is at equilibrium with a solution in which* 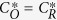
” and “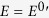 *and* 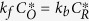
*, so that* 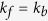
” for a simple electrochemical reaction (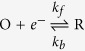), where 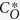 and 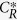 represent the concentrations of species O and R in the bulk, respectively, and 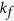 and 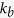
define the rate constants for the forward and backward reactions, respectively^65^. As previously discussed, this cannot be always assumed even for the simple electrochemical HER/HOR, but can only be assumed for much simpler reactions such as 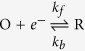. It follows that, for much more complicated reactions of ORR/OER, the applicability of the Butler-Volmer is questionable. We conclude that the Butler-Volmer equation cannot be *simply* applied to any reversible electrocatalytic reaction. It can be questioned to what extent evaluating the electrochemical reaction by the exchange current is valid. All electrochemical reactions must be evaluated based on the kinetics, not in conjunction with the Butler-Volmer equation. Therefore, the kinetics can be evaluated by the Tafel slope, which is kinetically always applicable, but *its intercept may not always be equal to the exchange current,* 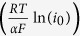.

### Other physical parameters associated with electrocatalytic activity

As described in this report, the electrocatalytic activity is not only potential-dependent but also temperature dependent, which alters the Tafel slope^107^,^108^,^109^. As well-known in the field of catalysis, the rate constant for the chemical reaction follows the Arrhenius’s equation:


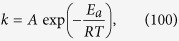


where *A* represents the collision frequency, and *E*_*a*_ represents the apparent activation energy. By varying the temperature during electrochemical measurement, the rate constants for each step can be altered, which in turn defines the Tafel slope. In the literature, Equation 3 is typically used to describe the dependence of the Tafel slope on temperature. In this equation, 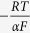 corresponds to the Tafel slope. This equation is derived based on the Butler-Volmer equation in conjunction with the Tafel equation, which is doubtful in our opinion, as discussed. Simply speaking, the Tafel slope indeed increases with elevated temperature^50^,^51^,^109^, but this increase likely arises from the increased rate constant. When the overall electric current is differentiated by potential, the detailed theoretical Tafel slope description is obtained. Further differentiating it by temperature reveals the dependence of the Tafel slope on temperature. This exercise is highly complicated and beyond the scope of this study.

The rate constant for the electrochemical reaction is generally given in Equation 10, where *α* is the transfer coefficient. The reference^52^ states that *“α, the transfer coefficient, can range from zero to unity”*. Because the transfer coefficient is included in the rate constant description, the Tafel slope is also dependent on the transfer coefficient. Experimentally identifying the transfer coefficient is quite difficult, but some have tried to determine it by directly correlating the transfer coefficient with the Tafel slope given by Equation 3 31,51 which is based on the assumption that the Butler-Volmer equation is applicable. The effort to experimentally elucidate the transfer coefficient should be made with considerable care, especially considering the applicability of the Butler-Volmer equation.

To accurately describe the kinetic component from overall current, the contribution of mass-transport must be effectively isolated. The following Levich equation has been established for the mass-transport limited current in the configuration of the rotating disk electrode (RDE)^52^:


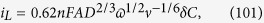


where *i*_*L*_ is the Levich currents (limiting diffusion current), *F* is the Faraday constant, *A* represents the electrode surface area, *ω* denotes the disk electrode rotation speed, *ν* is the kinematic viscosity and *δC* represents the difference between surface and bulk reactant concentrations. The relationship between the overall current *i* with the Levich current *i*_*L*_ and the kinetic current *i*_*k*_ is described in the Koutecky-Levich (KL) equation:


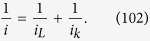


Using these equations, the kinetic current can be determined only when the system satisfies the assumptions required to establish the Levich equation. For example, a system consisting of considerable electrode roughness may lead to large deviations from the theoretical value simply expected from the KL equation. Thus, the KL analysis must be treated with special care based on these requirements. In general, the obtained values can be mostly overestimated, underestimated or even misled due to the multi-step nature of the reaction mechanism^110^. The considerations on mass-transport effects have been studied elsewhere for HER^111^,^112^,^113^, HER/HOR^4^,^112^,^113^ and ORR^114^ and are out of the scope of this study. However, it should be emphasized that improper subtraction of mass-transport contribution would cause misinterpretation of the rate determining steps and kinetics due to inaccurate Tafel slopes for elucidating kinetics^112^,^113^.

Microkinetic analyses are powerful for addressing reaction mechanisms and rate-determining steps. In addition, there is natural limitation in that the postulated mechanisms must be correct to describe the Tafel slopes. As observed in this study, the same Tafel slopes can be obtained for different elementary steps with varied coverages. For instance, in the OER section, the OER was described as a single-site mechanism, but recent advances in mechanistic studies of PSII in photosynthesis have proposed radical coupling mechanisms in which two metals are involved in the generation of one oxygen molecule^115^. In another example, it was proposed that HER via water reduction is facilitated by Ni hydroxide islands on Pt surfaces via a bifunctional mechanism at the periphery, where two different sites are involved in one reaction^4^,^116^,^117^. This study provides an aspect of electrocatalytic kinetics that focus on Tafel analyses and the applicability of the Butler-Volmer equation. The development of improved electrocatalysts should aim to identify catalysts that proceed via unexpected elementary steps, which breaks the volcano plot trend with one activity descriptor, such as metal-adsorbate bond strength^34^,^36^,^82^,^118^,^119^,^120^,^121^.

## Conclusions

Fundamental electrocatalytic reactions of hydrogen evolution reaction (HER), hydrogen oxidation reaction (HOR), oxygen reduction reaction (ORR) and oxygen evolution reaction (OER) were revisited considering conventional microkinetics and focusing on Tafel analyses. Our kinetic model reproduces the well-known Tafel slopes of 30, 40 and 120 mV dec^−1^ for the Tafel, Heyrovsky and Volmer steps of the HER, respectively, which confirms the validity of our method. Although in the literature, a Tafel slope of 120 mV dec^−1^ for the HER is generally assigned to the Volmer step, based on our analysis, this slope was also observed when the Heyrovsky step determined the rate with a high coverage of adsorbed hydrogen (>0.6). Similarly, a Tafel slope of 120 mV dec^−1^ was also found for the HOR, ORR and OER, but not only in the first discharge step as the rate-determining step. This observation suggests that a Tafel slope of 120 mV dec^−1^ (often considered as a single-electron transfer rate limiting) does not conclusively identify the rate-determining step. Furthermore, the validity of the Butler-Volmer equation to describe electrocatalytic kinetics in redox reactions was addressed in this study. Theoretical modeling suggests that only in very limited cases, where the electron transfer reaction determines the rate, is the Butler-Volmer equation applicable in describing the electrocatalytic kinetics. Therefore, the kinetics should be considered based on a microkinetic model that includes coverage terms rather than a model conjugated with the Butler-Volmer equation. Although the Tafel analysis is useful in elucidating the rate-determining steps, too simplified discussion, such as determination of the kinetics based only on the Butler-Volmer assumption, fails to accurately describe the surface electrocatalysis. This work provides a more accurate and concrete vision of the kinetics of H_2_ and O_2_ aqueous electrocatalysis, which is essential for the advancement of electrolysis and fuel cells.

## Additional Information

**How to cite this article**: Shinagawa, T. *et al.* Insight on Tafel slopes from a microkinetic analysis of aqueous electrocatalysis for energy conversion. *Sci. Rep.* **5**, 13801; doi: 10.1038/srep13801 (2015).
