# Author Correction: Insight on Tafel slopes from a microkinetic analysis of aqueous electrocatalysis for energy conversion

**DOI:** 10.1038/s41598-020-62993-x

**Published:** 2020-04-20

**Authors:** Tatsuya Shinagawa, Angel T. Garcia-Esparza, Kazuhiro Takanabe

**Affiliations:** Division of Physical Sciences and Engineering, KAUST Catalysis Center (KCC), King Abdullah University of Science and Technology (KAUST), 4700 KAUST, Thuwal, 23955-6900 Saudi Arabia

Correction to: *Scientific Reports* 10.1038/srep13801, published online 08 September 2015

In this Article, the authors did not originally include an assumption justifying why the activity of species, *a*_*i*_, (dimensionless) is used to describe the rates of the reactions. This is because these terms included standard concentration *c*^*0*^ (which is 1 M).

Therefore, in the Results and Discussion,

“The constants include Faraday’s constant, F = 96500 C mol^−1^, the gas constant, R = 8.314 J mol^−1^ K^−1^, temperature *T*, the electron transfer coefficient, α = 0.5, and the surface area of the electrode, A.”

should read:

“The constants include Faraday’s constant, F = 96500 C mol^−1^, the gas constant, R = 8.314 J mol^−1^ K^−1^, temperature *T*, the electron transfer coefficient, α = 0.5, and the surface area of the electrode, A. The activity of the species *i* is given by *a*_*i*_ = *γ*_i_*c*_i_/*c*°, where *γ*_i_ is the mean activity coefficient, *c*_i_ is the concentration, and *c*° is the standard concentration. Since the standard concentration is 1 M, the effective concentration can be simplified into *a*_*i*_*c*° = *a*_*i*_ and this is used hereafter.”

This change does not affect conclusions of the Article.

